# A Recurrent Case of Cryoglobulin-related Leukocytoclastic Vasculitis with an Unexpected Etiology

**DOI:** 10.7759/cureus.5783

**Published:** 2019-09-27

**Authors:** Anna Sudbury, Folake Akanbi, Radhika Kakarala, Ridhwi Mukerji

**Affiliations:** 1 Internal Medicine, Medical College of Wisconsin - Central Wisconsin, Wausau, USA; 2 Internal Medicine, McLaren Flint - Michigan State University, Flint, USA; 3 Hospital Medicine, Aspirus Riverview Hospital, Wisconsin Rapids , USA

**Keywords:** hep c, leukocytoclastic vasculitis, cryoglobulin, hepatitis c

## Abstract

We report a 64-year-old man presenting with cutaneous leukocytoclastic vasculitis, the underlying etiology of which was established as hepatitis C infection with associated cryoglobulinemia. This pathophysiologic state presented clinically as recurrent cutaneous vasculitic eruptions with the absence of any other clinical manifestations except for mild ankle swelling and weakness. This case clearly relates the need to consider hepatitis C as a potential etiologic factor in all patients with cutaneous vasculitis, and we suggest that viral hepatitis screening should be done routinely in all patients presenting with cutaneous vasculitis.

## Introduction

Chronic hepatitis C virus (HCV) is a major public health problem with a prevalence of 170 million people or nearly 3% of the world’s population [1-2]. However, more recent estimates of hepatitis C are much lower [3], with about 1.0% of the world’s population affected. This viral infection leads to a number of serious disorders, including liver cirrhosis, hepatocellular carcinoma, and extrahepatic manifestations that affect up to two-thirds of chronic HCV patients [2,4-5]. Some of these extrahepatic abnormalities include mixed cryoglobulinemia, glomerulonephritis, porphyria cutanea tarda, polyarticular arthritis, peripheral neuropathy, and various vasculitides. The strongest association exists with mixed cryoglobulinemia, as B lymphocyte expansion produces large amounts of circulating immune complexes, particularly rheumatoid factor with mixed cryoglobulins [4,6-7]. Around 40%-60% of patients with HCV have detectable serum cryoglobulins but only 10%-15% of those develop cryoglobulin-associated symptoms secondary to vascular occlusion and immune complex deposition with subsequent complement activation. The most common clinical manifestation is systemic vasculitis involving small and medium-sized blood vessels. We present a case of chronic hepatitis C with associated cryoglobulinemia manifesting as a cutaneous leukocytoclastic vasculitis in a seemingly healthy man.

## Case presentation

**Figure 1:**
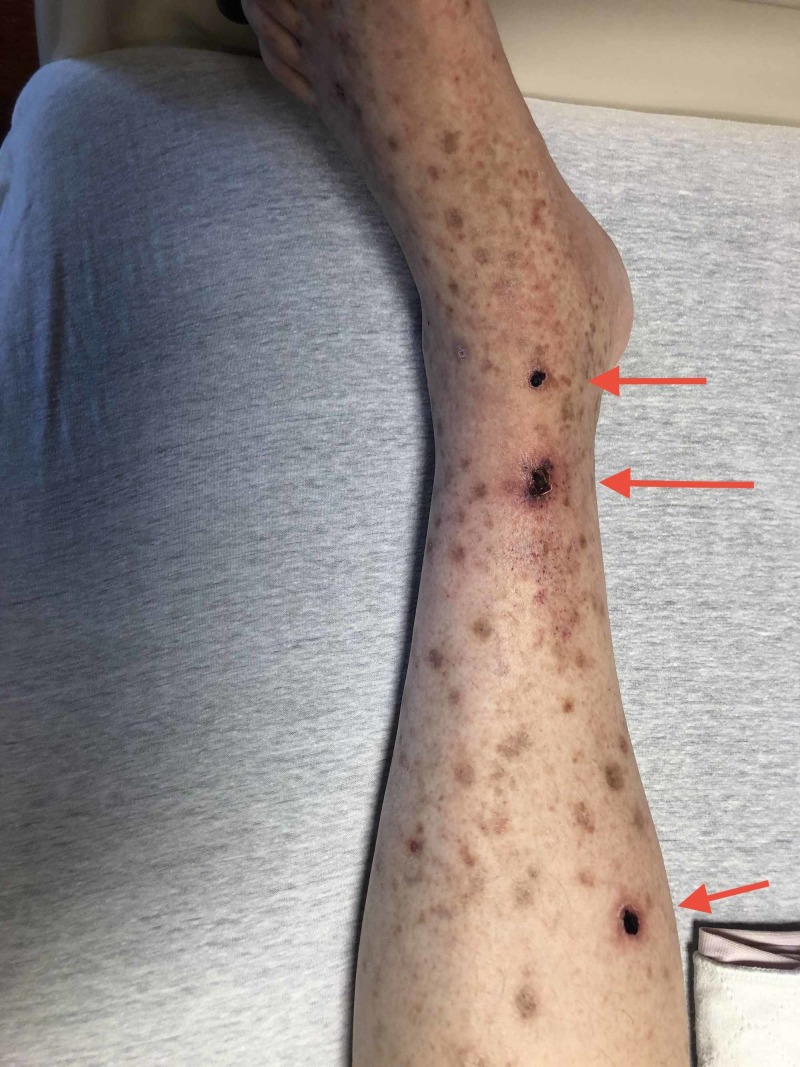
Lower extremity rash (left)

**Figure 2:**
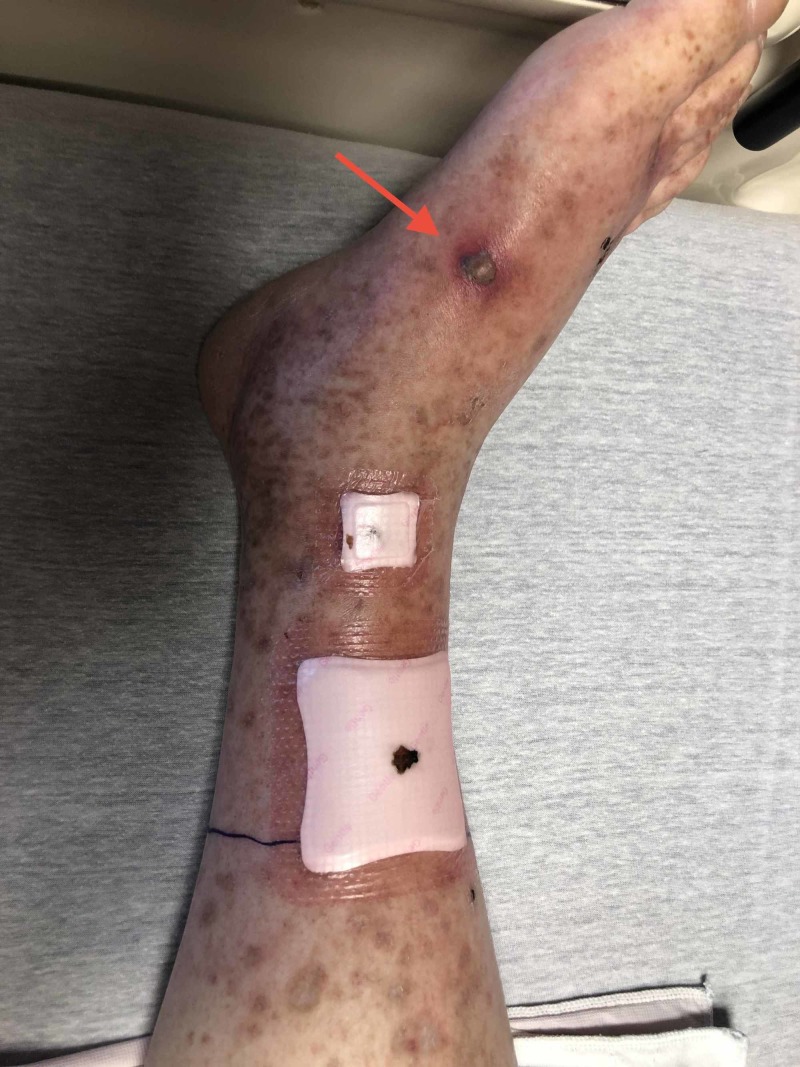
Lower extremity rash (right)

A 64-year-old man presented to our hospital with an episode of a skin rash and weakness involving his upper and lower limbs that had been recurring for the past four years (Figures 1-2). The patient reported that he lives alone in a house in the woods in Tennessee and visits Wisconsin often. He described recent progressive weakness due to pain and swelling in the right ankle and had great difficulty walking, even with a cane. He also reported generalized abdominal discomfort and reduced appetite. He has a history of hypothyroidism, and surgical history was unremarkable. He did not use alcohol or intravenous drugs but reported the use of marijuana and having a monogamous relationship with his ex-spouse. His only medication was 81 mg of aspirin daily. He denied recent infections and any new medication use.

Vital signs were within normal limits, except for elevated blood pressure readings of 178/96 mmHg and 196/105 mmHg measured one hour apart. The physical exam was noncontributory aside from a tender purpuric maculopapular rash predominantly present in the lower extremities. Laboratory studies are shown in Table 1.

**Table 1:** Laboratory values Normal ranges in parenthesis *Abnormal values are in bold

Labs	Values *
White blood cell count	4.8 (3.8 - 10.8 x10^3/uL)
Red blood cell count	3.74 (4.33-5.75x10^6/UL)
Hemoglobin	11.0 (13.4 -17.6 g/dL)
Hematocrit	32.7 (38.2-50.2%)
Mean corpuscular volume	87.4 (82.0 - 96.0 fL)
Mean corpuscular hemoglobin concentration	33.6 (32.4 - 35.7 g/dL)
Platelet count	204 (140-390 x10^3/uL)
D-Dimer	4.42 (0.15-0.49 ug/ml)
Glucose	111 (70-100 mg/dl)
Blood urea nitrogen	67 (8.0-24.0 mg/dl)
Creatinine	1.65 (.55-1.3 mg/dl)
Glomerular Filtration Rate calculation	43 (90-130 mls/min)
Sodium	142 (133-143 mEq/L)
Potassium	5.4 (3.5-5.0 mEq/L)
Chloride	108 (97-106 mEq/L)
Anion gap	12 (6-15 mmol/L)
Aspartate aminotransferase	42 (11-41 U/L)
Alanine aminotransferase	28 (11-66 U/L)
Alkaline phosphatase	29 (32-110 U/L)
Total bilirubin	0.5 (0-1.5 mg/dl)
Lactic acid	0.8 (0.4-1.9 mmol/L)
Pro-B-natriuretic peptide	434 (0-300 pg/ml)
Total protein	6.0 (6.2-8.5 g/dl)
Albumin	3.4 (3.5-4.2)
Thyroid stimulating hormone	20.67 (.60-5.40 ulU/ml)
Free T4	1.01 (.0-1.5 ng/dl)
Urine color	dark yellow (yellow)
Urine appearance	clear (clear)
Urine pH	5.0 (5.0-8.0)
Urine specific gravity	>=1.030 (1.010 - 1.030)
Urine protein	3+ (negative)
Urine ketones	Negative (negative)
Urine blood	Large (negative)
Urine nitrite	Negative (negative)
Urine bilirubin	Small (negative)
Urine urobilinogen	.2 (.2-1.0 EU/dL)
Urine leukocyte esterase	Negative (negative)
Urine red blood cell count	11-20 (0-2/hpf)
Urine white blood cell count	0-1 (0-2/hpf)
Urine epithelial cells	2-5 (0-10/hpf)
Urine bacteria	Trace (none)
Urine glucose	Negative (negative)
Urine drug screen	Marijuana (negative)
Lyme total antibody	Negative (negative)
Borrelia burgodorferi antibody index	Negative (negative)
Chlamydia	Negative (negative)
Gonorrhea	Negative (negative)

The complete metabolic panel showed hyperkalemia, hyperchloremia, elevated blood urea nitrogen (BUN) and creatinine, decreased glomerular filtration rate (GFR), mildly elevated aspartate aminotransferase (AST), pro-B-natriuretic peptide (Pro-BNP), and thyroid-stimulating hormone (TSH), as well as reduced total protein and albumin. D-Dimer was found to be elevated. The complete blood cell count revealed mild anemia with no other abnormalities. Lyme total antibody and Borrelia burgdorferi antibodies were negative. Chlamydia and gonorrhea urine polymerase chain reaction (PCR) tests were negative. Urinalysis showed high urine specific gravity, positive urine for protein, large amounts of blood, and high red blood cell (RBC) count suggestive of glomerulonephritis. The urine drug screen was positive for marijuana only. Kidney biopsy showed membranoproliferative glomerulonephritis.

Inflammatory markers and an autoimmune screen, which included myeloperoxidase antibodies, serine protease 3 antibodies, double-stranded deoxyribonucleic acid (DNA), antinuclear antibodies (ANA), were negative and remained so on repeat sampling. The patient also had normal anti-streptolysin O titer, complement, and immunoglobulin levels. Hepatitis A and B and human immunodeficiency virus (HIV) testing were negative, and hepatitis C serology was positive. Chronic hepatitis C virus infection was diagnosed by enzyme immunoassay (anti-HCV antibody test) and confirmed by a reverse-transcriptase polymerase chain reaction, which showed a ribonucleic acid (RNA) burden of 1.9 million IU/L. As a result of the patient’s rash and presence of HCV, cryoglobulin IgG, IgA, and IgM were tested and were elevated. The biopsy of the lesions revealed leukocytoclastic vasculitis. Given the association between hepatitis C infection and type II mixed cryoglobulinemia, a diagnosis of hepatitis C-related cryoglobulinemic cutaneous vasculitis was made.

Abdominal imaging was obtained because of abdominal discomfort, and it showed a cirrhotic liver and splenomegaly. Serpiginous densities adjacent to the distal esophagus were compatible with periesophageal varices, with thickening of the visualized distal esophagus. Venous Doppler studies of the lower extremities were obtained because of the patient’s lower extremity swelling and were negative for deep venous thrombosis (DVT).

He was referred to a hepatologist, nephrologist, and general practitioner for the management of chronic hepatitis C infection, membranoproliferative glomerulonephritis, cryoglobulin-related leukocytoclastic vasculitis, and hypertension. The patient denied all known risk factors of HCV and the source of hepatitis C infection remained unknown.

## Discussion

We hypothesized several etiological mechanisms, including autoimmune disease, infectious processes, and intrinsic renal abnormalities, because of the initial presentation of weakness and rash coupled with hypertension. The differential diagnoses included systemic lupus erythematosus, Goodpasture disease, granulomatosis with polyangiitis, microscopic poly-angiitis, sexually transmitted infections, and Lyme disease in view of the season and residence in northern Wisconsin and Tennessee.

At the time of initial presentation to the inpatient service, the clinical suspicion of chronic hepatitis C infection in this otherwise healthy man was low. Our patient presented with palpable purpura, which is a cutaneous manifestation of underlying hepatitis C infection; other cutaneous manifestations include livedo reticularis, lichen planus, erythema multiforme and nodosum, and porphyria cutanea tarda [8-10]. Acute hepatitis C infection is usually asymptomatic as is chronic hepatitis C infection, with only 20% of individuals progressing to cirrhosis within 10-30 years [11].

Extensive autoimmune and antibody testing lead to the clinical suspicion of cryoglobulin-related leukocytoclastic vasculitis. Cryoglobulins are immunoglobulins that exhibit precipitation at temperatures below 37°C, return to a solutional state with warming, and can cause abnormalities in the vasculature. They may precipitate and obstruct small peripheral vessels resulting in ischemia and infarction, or they may deposit in the vasculature as immune complexes and cause leukocytoclastic vasculitis. This vasculitis may present in multiple ways, such as peripheral neuropathy, or as seen in our patient, renal damage resulting in glomerulonephritis and palpable purpura. Further hematological associations include lymphoma and myeloma, and it also co-exists and is related to autoimmune disorders such as rheumatoid arthritis and systemic lupus erythematosus. Infections implicated in the causation of cryoglobulinemia include parasitic, bacterial, and viral infections, of which HCV is more common than hepatitis B virus. Type 2 mixed cryoglobulinemia is characterized by polyclonal immunoglobulin G (IgG) and monoclonal IgM rheumatoid factor directed against the IgG component whereas in type 3, both the IgG and IgM rheumatoid factors, are polyclonal. Hepatitis C infection may be responsible for a majority of cases of non-primary cryoglobulinemia, as anti-HCV antibodies are found in greater than 70% of patients with mixed cryoglobulinemia [12-13].

Mixed cryoglobulins are present in the course of connective tissue and autoimmune diseases and in chronic infections. Arthralgias more frequently involve the hands and knees symmetrically, although, in our patient, it manifested in the ankles. Weakness is nearly always present, and the kidney, liver, and nervous system are frequently involved [7]. Renal injury secondary to cryoglobulins may complicate the diagnosis, as it is present in almost 30% of cases of cryoglobulinemia and in 20% of whom nephropathy is present at diagnosis [14-15]. Hypertension, proteinuria, microhematuria, red blood cell casts, and renal failure are present in about 50% of cases while nephritic and nephrotic syndromes are less common (14% and 21% respectively) [7,14].

The definitive management of hepatitis C-related cryoglobulinemia is the eradication of the virus with antiviral treatment and the suppression of the cryoglobulinemic process via the use of antivirals and systemic glucocorticoids in combination with rituximab or cyclophosphamide [16-17]. Additional regimens include pegylated interferon alpha 2a or 2b in combination with antivirals such as ledipasvir/sofosbuvir [18-19]. Current treatment options for hepatitis C infection provide cure with an eight to 12-week course and have dramatically changed the natural history of this infection [20].

## Conclusions

In summary, our case report relates the association between hepatitis C virus, cryoglobulinemia, and leukocytoclastic vasculitis. Providers should be aware of the cutaneous and other manifestations of hepatitis C, as it is largely asymptomatic, and these might be the only manifestations present for this largely silent but drastic infection. Cutaneous vasculitis is a relatively common clinical presentation of hepatitis C, and this case emphasizes the need to maintain a broad differential even in patients with no apparent risk factors. In view of the possibility of a cure for this deadly infection, a high index of suspicion, thorough history taking, physical examination, and investigation is required to facilitate prompt diagnosis and treatment.
